# Conflict in ant phylogeny results from complex interaction between multiple evolutionary signals and tree reconstruction artifacts

**DOI:** 10.1093/molbev/msag058

**Published:** 2026-03-07

**Authors:** Wentao Tao, Giorgio Bianchini, Enrico Schifani, Christopher Kay, Donato A Grasso, Philip C J Donoghue, Davide Pisani

**Affiliations:** Bristol Palaeobiology Group, Schools of Biological Sciences, University of Bristol, Life Sciences Building, Bristol BS8 1TQ, UK; School of Geographical Sciences, University of Bristol, Bristol BS8 1SS, UK; Institute of Evolutionary Biology (CSIC—Pompeu Fabra University), Passeig Marítim de la Barceloneta, 37-49, 08003 Barcelona, Spain; Bristol Palaeobiology Group, Schools of Biological Sciences, University of Bristol, Life Sciences Building, Bristol BS8 1TQ, UK; Department of Chemistry, Life Sciences and Environmental Sustainability, University of Parma, Parco Area delle Scienze 11/a, Parma 43124, Italy; Bristol Palaeobiology Group, School of Earth Sciences, University of Bristol, Life Science Building, Bristol BS8 1TQ, UK; Bristol Palaeobiology Group, Schools of Biological Sciences, University of Bristol, Life Sciences Building, Bristol BS8 1TQ, UK

**Keywords:** Mixture models, phylogenetics, CAT-PMSF, Formicidae, Martialinae

## Abstract

Ants, Formicidae, are a group of small social insects that inhabit nearly all terrestrial environments. Three competing hypotheses of ant relationships have been proposed, differing in the placement of Martialinae, a subfamily of cryptic, endogean ants. We used BUSCO genes to investigate the signals in individual and concatenated gene datasets. We found that gene trees support all three hypotheses. After concatenation, the three signals persist but their relative strength is model-dependent. The CAT-posterior mean site frequencies approach (which our model adequacy tests show best explains the across-site compositional heterogeneity of the data) finds Martialinae as the sister of all ants but Leptanillinae. We tested the effect of across-lineage compositional heterogeneity using data-recoding and excluding highly heterogeneous taxa. These tests did not lead to the emergence of significant support for alternative tree topologies. However, we identified strong gene- and site-discordance in the data and evidence that signals representing incongruent evolutionary processes exist in ant genomes supporting all three hypotheses. Incomplete lineage sorting and/or introgression seem to have significantly affected early ant evolution, which might make it impossible to establish whether Leptanillinae, Leptanillinae plus Martialinae, or Martialinae represents the sister of all the other ants.

## Introduction

Ants are one of the most diverse insect lineages, comprised of more than 14,000 living species that thrive in almost all terrestrial ecosystems (Economo et al. 2018). The ecological importance and eusocial behaviors of ants have driven extensive research to understand their phylogenetic relationships (Brady et al. 2006; Moreau et al. 2006; Moreau and Bell 2013; Ward 2014; Branstetter et al. 2017; Chomicki and Renner 2017; Nelsen et al. 2018; Borowiec et al. 2019; Romiguier et al. 2022; Cai 2024; Borowiec et al. 2025; Vizueta et al. 2025). However, disentangling the relationships of the ant lineages stemming close to the root of the ant phylogeny has proven difficult (Borowiec et al. 2019; Romiguier et al. 2022; Cai 2024, 2025; Boudinot and Lieberman 2025).

Formicidae can be split into three major lineages: the leptanilloid, poneroid and formicoid clades (Brady et al. 2006; Moreau et al. 2006). The leptanilloid clade includes the subfamily Leptanillinae that was assumed to represent the sister-group of all the other living ants (hereafter Leptanillinae–sister hypothesis) until the discovery of *Martialis heureka* (Rabeling et al. 2008). *Martialis heureka* is a cryptic hypogaeic forager, likes Leptanillinae, and shares with this lineage a diversity of morphological characters, such as the absence of compound eyes and frontal carinae in workers (Boudinot 2015). Rabeling et al. (2008) integrated *M. heureka* into the seven nuclear gene dataset of Brady et al. (2006), concluding that *M. heureka* was the sister of all other ants (hereafter Martialinae–sister hypothesis). However, with a Maximum Likelihood (ML) Bootstrap (BP) of 76 and a Bayesian Posterior Probability (PP) of 0.91 (Rabeling et al. 2008), this result was only moderately supported. Kück et al. (2011) performed new analyses of the Rabeling et al. (2008) dataset, implementing new alignment masking protocols, reverting support to the Leptanillinae–sister hypothesis, with Martialinae representing the sister of all the ants but Leptanillinae. Leptanillinae–sister has received further support from some subsequent studies (eg Moreau and Bell 2013; Ward and Fisher 2016; Cai 2024). Borowiec et al. (2019) expanded over previous studies collecting 11 nuclear loci across 110 ant species and performing sophisticated analyses to account for the potentially negative impact of across-lineage compositional heterogeneity on ant phylogeny. These authors recovered a third tree topology where Leptanillinae and Martialinae form a clade (also known as Leptanillomorpha, see Oberski et al. 2025) that is sister to all the other ants (hereafter, the Leptanillinae plus Martialinae–sister hypothesis). Following the recent development of target-enrichment and next-generation sequencing techniques, ultraconserved elements (UCEs) and genome-scale superalignments of protein-coding genes were used to test hypotheses of ant relationships. Romiguier et al. (2022) used 4,151 single-copy protein-coding genes (1,704,506 amino acid positions) from 82 taxa and found support for Leptanillinae plus Martialinae–sister using a C20-based (PMSF-LG + F + G + C20) across-site compositionally heterogeneous empirical mixture model with fixed numbers of site-frequency categories (Le et al. 2008). Borowiec et al. (2025) confirmed these results using a UCE dataset from which they generated nucleotide and amino acid alignments that they analyzed using both across-site compositionally homogeneous and heterogeneous models. Similarly to Romiguier et al. (2022) also Borowiec et al. (2025) used a C20-based, across-site compositionally heterogeneous, model. However, Cai (2024) performed reanalyses of the amino acid dataset of Romiguier et al. (2022) using an across-site compositionally heterogeneous infinite mixture model (CAT-GTR + G), finding support for Leptanillinae–sister.

Giacomelli et al. (2022) performed simulations showing that in analyses of amino acid data, CAT-GTR + G accommodates across-site compositional heterogeneity better than both compositionally homogeneous models such as GTR and LG, and across-site compositionally heterogeneous models with fixed numbers of site-frequency categories (see Giacomelli et al. (2022); Fig. 2c). That is, the CAT-GTR + G model of Cai (2024) should be expected to describe the across-site compositional heterogeneity in the data better than the models used in previous studies. However, Boudinot and Lieberman (2025) raised a number of concerns with the study of Cai (2024), ranging from the use of an alignment curation protocol that was too aggressive, to the use of a model that was too complex and the possibility that Cai (2024) may not have run their analyses to convergence. Principally, Boudinot and Lieberman (2025) argued that the CAT-GTR + G model used by Cai (2024) does not account for compositional heterogeneity, but only by redefining compositional heterogeneity to encompass only variation in amino acid preferences observed across lineage. However, by doing so, Boudinot and Lieberman (2025) overstated their case. Cai (2024) did not account for across-lineage compositional heterogeneity, but it did not claim to do so (see also Cai 2025), as it focused on across-site compositional heterogeneity. To avoid confusion, we are explicit in qualifying what form of compositional heterogeneity we refer to, distinguishing between across-site and across-lineage (or taxa) compositional heterogeneity. In all instances where we do not explicitly state whether we are referring to heterogeneity acting across taxa or sites, the reader should assume that we are referring to both forms of heterogeneity at the same time.

Compositional heterogeneity is not the only factor that can account for incongruence in ant phylogenies. Other possible factors are introgression and incomplete lineage sorting. In an attempt to resolve the phylogenetic controversy surrounding the placement of Martialinae, we assembled a new genome-scale dataset comprising 1,442 BUSCO genes (1,105,819 amino acid positions) and tested competing hypotheses of ant relationships. We took advantage of recent improvements in studying gene tree incongruence (Lanfear and Hahn 2024) and the newly developed CAT-posterior mean site frequencies (PMSF) procedure (Szánthó et al. 2023) which, in an ML framework, is expected to account for across-site compositional heterogeneity better than empirical mixture models with fixed number of site-frequency categories (eg Giacomelli et al. 2024). We also used approaches that reduce across-lineage compositional heterogeneity: data recoding (Feuda et al. 2017; Giacomelli et al. 2022) and heterogeneous taxa deletion experiments (Borowiec et al. 2019).

We found that there are three signals in ant genomes, representing genuine but incongruent evolutionary processes, that correspond to the three competing hypotheses of ant relationships (Leptanillinae–sister, Leptanillinae plus Martialinae–sister, and Martialinae–sister). Once the effect of compositional heterogeneity across both lineages and sites is accounted for, the Leptanillinae–sister signal is strongest. However, the approximately unbiased (AU) test could not discriminate between alternative hypotheses of ant relationships, suggesting that the difference in magnitude between these signals is marginal, while results of gene and site concordance analyses suggest that incomplete lineage sorting and/or introgression, seem to have had an impact on early ant evolution. Accordingly, we conclude that neither our results nor those of previous studies can provide the evidence necessary to conclude which one of these signals (irrespective of their relative strength) is representative of the true ant phylogeny, which may not be resolvable in any meaningful sense if early ant evolution was dominated by introgressive events.

## Results

### Datasets

We started from a dataset including 101-taxon and 1,442 genes (1,105,819 amino acid positions), see Materials and Methods for details. Despite our dataset can be easily analyzed using fast heuristic approaches such as ASTRAL, it cannot be analyzed as a concatenated superalignment using the infinite mixture models accounting for across-site compositional heterogeneity that have featured prominently in recent debate over ant phylogeny (Cai 2024; Boudinot and Lieberman 2025). Accordingly, we generated a second dataset including 55 taxa that we used for all the analyses that used mixture models accounting for across-site compositional heterogeneity over a concatenated dataset. To make sure that taxon subsampling did not distort the signal in our dataset, we started performing two astral analyses, one for the 101-taxon dataset and one for the 55-taxon dataset and tested whether they converged on the same topology.

The two ASTRAL analyses (101-taxon and 55-taxon datasets) inferred the same tree topology, both supporting the Leptanillinae plus Martialinae–sister hypothesis (Fig. S1). The only difference between these trees relates to the placement of *Camponotus floridanus,* a formicoid species that is distantly related to the lineages representing the focus of our study. We therefore conclude that subsampling the 101-taxon dataset to 55 taxa did not affect its phylogenetic signal in a way that is a significant to the problem we are addressing in this study. Accordingly, all subsequent analyses used the 55-taxon dataset.

### The Leptanillinae plus Martialinae–sister hypothesis is preferred by more gene trees

We tested whether the 1,442 gene alignments best-fit one of the across-site compositionally homogeneous or one of the across-site compositionally heterogeneous models available in IQTREE. We found that 1,388 alignments were best-fit to an across-site compositionally heterogeneous empirical mixture model with a fixed number of categories (ie LG + F + G + CXX models—where XX can be 10, 20, …, 60). The remaining 54 alignments were best-fit to an across-site compositionally homogeneous model (eg LG + F + G or LG + F + R models) (Table S1). The phylogenetic hypothesis most frequently supported in single-gene analyses was Leptanillinae plus Martialinae–sister. This hypothesis is supported by 574 BUSCO genes (Table S2) and the gene concordance factor (gCF) and site concordance factor (sCF) for a phylogeny resolving Leptanillinae plus Martialinae–sister are gCF = 39.8 and sCF = 35.1 (Fig. S2a). Leptanillinae–sister is the second most frequently supported hypothesis, recovered from the analysis of 413 gene trees (Table S2). The gCF and sCF for an ant phylogeny resolving Leptanillinae–sister is gCF = 28.6 and sCF = 33.7 (Fig. S2b). Martialinae–sister emerges as the least frequently supported topology, resolved by 331 gene trees (gCF = 23.0, sCF = 31.1; Table S2 and Fig. S2c). The difference in the number of trees supporting alternative hypotheses of ant relationships is significant (χ^2^ = 69.571, df = 2, *P*-value = 7.815e−16), even if we take into consideration that 124 genes (8.6% of the gene trees) do not support any of the three considered hypotheses (χ^2^ = 100.05, df = 2, *P*-value < 2.2e−16). However, gCF and particularly sCF values are close to the values expected if the ant phylogeny was polytomous (which would be 33%). We note that 211 of the 1,442 genes find Leptanillinae paraphyletic. Excluding these genes leave us with 1,231 genes, of which 1,163 support one of the three alternative topologies: 505 (43.4%) support Leptanillinae plus Martialinae–sisters, 359 (30.9%) support Leptanillinae–sister, and 299 (25.7%) support Martialinae–sister. These proportions closely match those obtained when these genes are included: 43.6% for Leptanillinae plus Martialinae–sisters, 31.3% for Leptanillinae–sisters, and 25.1% for Martialinae–sisters, indicating that the inclusion (or exclusion) of these genes does not alter our conclusions.

The signal in single-gene alignments might lack the power to discriminate between alternative tree topologies (eg Tarver et al. 2016) and a fraction of individual gene trees might be erroneous (Xi et al. 2015) and agree with a given hypothesis of ant phylogeny just by chance. Hence, we performed AU tests for each of the 1,442 genes (under their best-fitting model) and found that only 35 of them (2.4%) can statistically discriminate between the three competing hypotheses (Table S3). Of these 35 genes, 16 support Leptanillinae plus Martialinae–sister, 16 Leptanillinae–sister and 3 Martialinae–sister.

### CAT-PMSF + G describes across-site compositional heterogeneity significantly better than LG + G and LG + F + G + C60

We used parametric bootstrap to test which model best describes the across-site compositional heterogeneity of ten jackknifed datasets subsampled from the full 1,442-gene superalignment (see Materials and Methods for details). The measure we used to test fit was the mean amino acid diversity per site (div), the same statistic used in Phylobayes MPI v1.9 (see Phylobayes manual for details) to achieve the same goal using Posterior Predictive Analysis (PPA) (Giacomelli et al. 2024).

**Figure 1:**
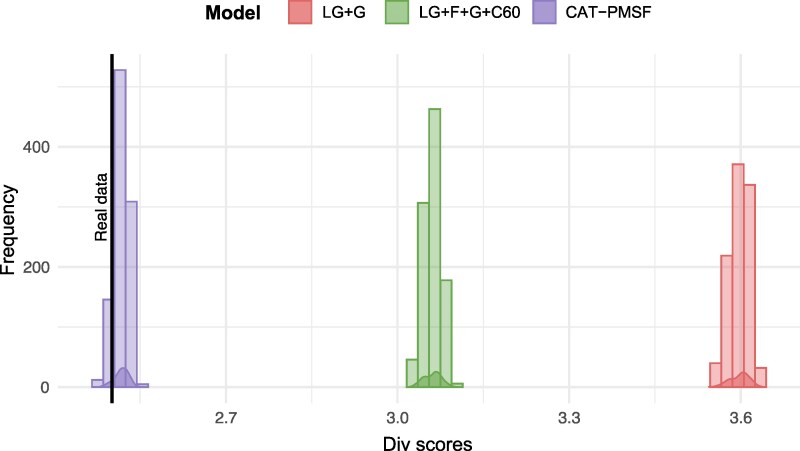
Results of the parametric bootstrap based model adequacy tests. The figure presents distribution of div scores for datasets simulated using parametric bootstrap under LG + G, LG + F + G + C60, and CAT-PMSF + G. The figure summarise distributions for datasets simulated using models trained on each of the 10 jackknife datasets (1,000 samples in total – 100 points for each dataset). The black line indicates the average div score observed for the real data. When the distribution of simulated datasets, for a model, includes the value for the real data, the model adequately describes (ie fits) the data. The further the distribution is from the value representing the real data the worse is the fit of the model to the data.

The results of the parametric bootstrap analyses are shown in Fig. 1. *Z*-scores and the distribution of div scores are also presented in Tables S4 and S5. These tests clearly show that LG + G and LG + F + G + C60 provide a very poor description of the data (*Z*_LG_ = 100.14–118.84, with an average of 112.16, and *Z*_LG + F + G + C60_ = 42.24–50.46, with an average of 46.84), despite LG + F + G + C60 doing better than LG + G (as expected) across the ten jackknifed datasets. On the contrary, *Z*_CAT-PMSF_ = 0.75–4.79 (Average = 2.69) indicates that CAT-PMSF + G describes the data significantly better than LG + G and LG + F + G + C60.

### Single-signal datasets always support their own topology

Results of analyses of single-signal datasets, the superalignments concatenating exclusively genes that in single gene analyses supported the same hypothesis for the relationships of *M. heureka*, are model-independent and always support the same topology supported by the concatenated genes when independently analysed. Our parametric bootstrap analyses demonstrate that CAT-PMSF + G best describes the across-site compositional heterogeneity of these datasets (Tables S4 and S5), average *Z*_LG_ = 105.72, average *Z*_LG + F + G + C60_ = 48.34, and average *Z*_CAT-PMSF_ = 3.04. Under CAT-PMSF + G, the model that best describes the across-site compositional heterogeneity of these datasets, the Leptanillinae plus Martialinae–superalignment supports Leptanillinae plus Martialinae–sister with Composite Ultrafast Bootstrap Support values (CUBS; see Methods for details) = 72, the Leptanillinae–superalignment supports Leptanillinae–sister with CUBS = 100, and the Martialinae–superalignment supports Martialinae–sister with CUBS = 96. The Leptanillinae–superalignment, despite being based on concatenation of fewer genes than the Leptanillinae plus Martialinae–superalignment, seems to convey a more consistent signal; it is the only dataset providing full CUBS to its own phylogeny and it is the only one that can significantly discriminate between alternative hypotheses of ant relationships using AU tests (see Table S6).

### CAT-PMSF + G analyses of our concatenated dataset prefers the Leptanillinae–sister hypothesis

**Figure 2:**
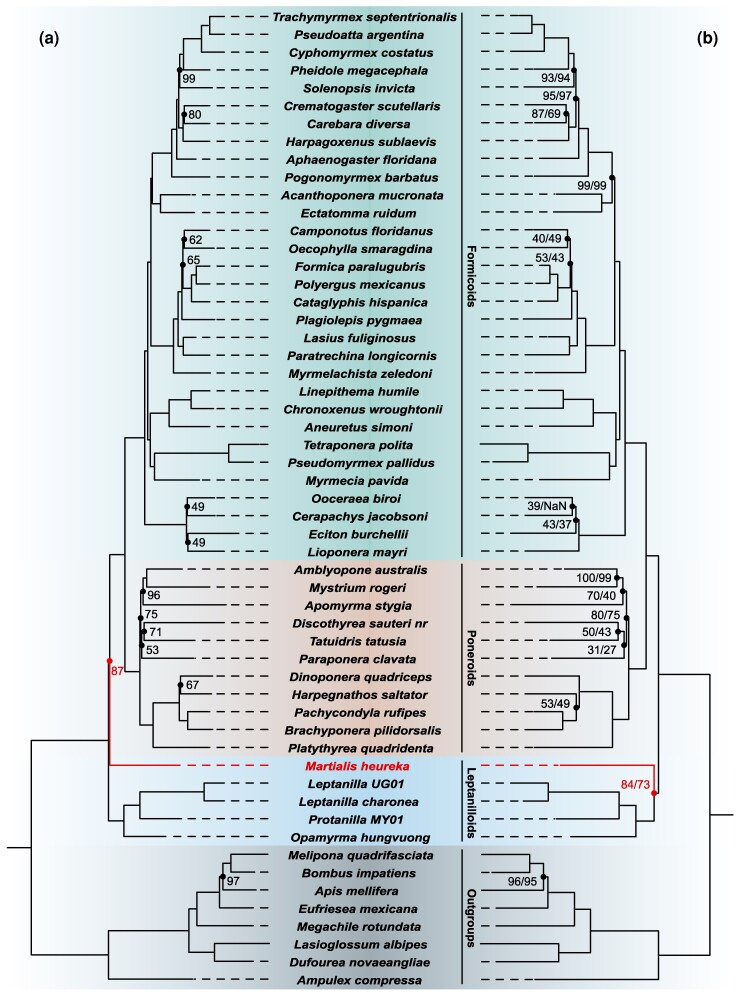
Comparison of bootstrap consensus trees representing the summary of ultrafast bootstrap trees pooled for all ten jackknife datasets (ie Cumulative Bootstrap Support–CUB–values) under CAT-PMSF + G, LG + G, and LG + F + G + C60. (a) Bootstrap consensus tree summarising 30 CAT–PMSF + G analyses (see text and Methods for details). (b) Bootstrap consensus tree of ten phylogenetic analyses under LG + G and LG + F + G + C60 (Methods for details). The LG + G and LG + F + G + C60 analyses inferred the same tree. When two CUBS are presented in panel b, the first represents support under LG + F + G + C60 and the second indicates support under LG + G.

We performed phylogenetic analyses of ten jackknifed datasets (see Methods for details), generated subsampling the concatenated alignment of all considered genes, irrespective of the phylogeny that they supported when independently analysed. Under LG + G and when the best fitting among the empirical mixture model with a fixed number of categories (LG + F + G + C60) was used, these datasets provided overall support for the Leptanillinae plus Martialinae–sister hypothesis (Table 1 and Fig. 2—respectively with CUBS = 73 and 84). However, if we look at individual Jackknifed datasets, we find that while most analyses support Leptanillinae plus Martialinae–sister, there are three exceptions. These are the LG + G analysis of jackknifed dataset 1 and the LG + G and LG + F + G + C60 analyses of jackknifed dataset 7, all of which support Martialinae–sister. Under LG + G and LG + F + G + C60, Leptanillinae–sister is never recovered. Differently, under CAT-PMSF + G, support is invariably found for Leptanillinae–sister. In agreement with Giacomelli et al. (2024), we found that the tree used in Phylobayes to infer the site-frequency profiles used for the CAT-PMSF analyses had some impact on the support provided to alternative hypotheses of ant relationships, with the support for Leptanillinae–sister being lower when the site-frequency profile was inferred on a tree displaying either Leptanillinae plus Martialinae–sister or Martialinae–sister. Nonetheless, all CAT-PMSF + G analyses supported Leptanillinae–sister (CUBS = 87; Table 1 and Fig. 2).

**Table 1:** Ultrafast bootstrap values or posterior probabilities and supported topologies across different datasets and models for our amino acid and Dayhoff-6 recoded datasets.

Dataset	Amino acids												Dayhoff-6			
	LG + G		LG + G + F + C60		CAT-PMSF + G						GTR + G		GTR + G		CAT-Poisson + G	
					Mar–sister		Lep–sister		Lep + Mar–sister							
	Topology	UFB	Topology	UFB	Topology	UFB	Topology	UFB	Topology	UFB	Topology	PP	Topology	PP	Topology	PP
JK0	Lep + Mar	84	Lep + Mar	90	Lep	92	Lep	91	Lep	85	Lep + Mar	1	Lep	0.99	Lep	0.56
JK1	Mar	74	Lep + Mar	76	Lep	90	Lep	91	Lep	89	Mar	1	Lep + Mar	1	Lep	0.63
JK2	Lep + Mar	96	Lep + Mar	97	Lep	82	Lep	92	Lep	85	Lep + Mar	1	Lep + Mar	1	Lep	0.8
JK3	Lep + Mar	79	Lep + Mar	82	Lep	91	Lep	96	Lep	91	Lep + Mar	0.97	Lep + Mar	1	Lep	0.96
JK4	Lep + Mar	80	Lep + Mar	93	Lep	92	Lep	96	Lep	94	Lep + Mar	1	Lep + Mar	0.94	Lep	0.99
JK5	Lep + Mar	94	Lep + Mar	100	Lep	87	Lep	93	Lep	86	Lep + Mar	1	Lep + Mar	1	Lep + Mar	0.84
JK6	Lep + Mar	91	Lep + Mar	97	Lep	76	Lep	79	Lep	66	Lep + Mar	1	Lep + Mar	1	Lep	0.8
JK7	Mar	97	Mar	56	Lep	87	Lep	97	Lep	93	Mar	1	Lep	0.99	Lep	0.99
JK8	Lep + Mar	97	Lep + Mar	97	Lep	74	Lep	83	Lep	81	Lep + Mar	1	Lep + Mar	1	Lep	0.47
JK9	Lep + Mar	84	Lep + Mar	85	Lep	93	Lep	97	Lep	93	Lep + Mar	1	Lep	0.99	Lep	0.93

The tree used to infer the CAT-PMSF models are indicated as: Mar–sister, Martialinae–sister; Lep–sister, Leptanillinae–sister; Lep + Mar–sister, Leptanillinae plus Martialinae–sister.

AU tests were performed on each of the ten jackknife datasets under each of the three CAT-PMSF + G models (Table S6). The results confirm that under CAT-PMSF + G, Leptanillinae–sister is the preferred hypothesis (Average AU *P*-value_Leptanillinae–sister_ = 0.838). However, the two alternative hypotheses are never rejected (Average *P*-value_Leptanillinae plus Martialinae–sister_ = 0.171; Average *P*-value_Martialinae–sister_ = 0.162).

### Across-lineage compositional heterogeneity affects only on our concatenated datasets

We investigated whether our single-gene alignments were affected by across-lineage compositional heterogeneity. We found that only 140 out of 1,442 gene alignments (∼9.7%) included taxa with this kind of heterogeneous amino acid composition (Table S7). In all cases, only a few taxa were heterogeneous. To put things in perspective, the most heterogeneous (across-lineage) of our gene alignments (BUSCO gene 1895at7399) includes 16 (out of 55) compositionally heterogeneous taxa. Furthermore, in only 8 of the 140 heterogeneous single-gene alignments, *M. heureka* is one of the compositionally heterogeneous taxa. Four of the eight trees where *M. heureka* genes are compositionally heterogeneous support Leptanillinae plus Martialinae–sister, three support Leptanillinae–sister, and only one supports Martialinae–sister. Similarly, only 20 of 140 single-gene alignments include compositionally heterogeneous Leptanillinae. Of these 20 genes, 9 supported Leptanillinae plus Martialinae–sister, 7 support Leptanillinae–sister, and 1 supported Martialinae–sister.

In the 10 jackknifed (concatenated) datasets, 9 to 13 taxa failed the χ^2^ test of compositional heterogeneity across taxa. Many of these taxa belong to the outgroup or Leptanillinae (*Leptanilla_UG01*, *Leptanilla_charonea*, and most often *Protanilla_MY01*). We excluded heterogeneous taxa from the ten original jackknifed datasets and inferred new trees under LG + G, LG + F + G + C60, and CAT-PMSF + G (Table 2). We found that after removing all compositional heterogeneous taxa, the previously weak support (found in 3 analyses out of 20) for Martialinae–sister (under LG + G and LG + F + G + C60—see also above) disappeared, giving way to support for Leptanillinae plus Martialinae–sister. Meanwhile, 2 out of the 20 analyses that previously supported Leptanillinae plus Martialinae–sister shift support to Leptanillinae–sister. In the CAT-PMSF + G analyses, the Leptanillinae–sister hypothesis remained preferred but with reduced support, with 3 of the 30 analyses shifting support to Martialinae–sister.

**Table 2:** Ultrafast bootstrap values and supported topologies across different datasets and models after removal of compositionally heterogeneous taxa.

Taxon removal datasets	LG + G		LG + F + G+ C60		CAT-PMSF + G					
					Mar–sister		Lep–sister		Lep + Mar–sister	
	Topology	UFB	Topology	UFB	Topology	UFB	Topology	UFB	Topology	UFB
JK0(5/1)	Lep + Mar	98	Lep + Mar	100	Lep	73	Lep	84	Lep	64
JK1(3/1)	Lep + Mar	79	Lep + Mar	99	Mar	48	Lep	48	Lep	38
JK2(5/1)	Lep + Mar	98	Lep + Mar	100	Lep	44	Lep	58	Lep	42
JK3(4/2)	Lep + Mar	87	Lep + Mar	95	Lep	70	Lep	82	Lep	59
JK4(3/1)	Lep + Mar	88	Lep + Mar	99	Lep	50	Lep	72	Lep	63
JK5(2/1)	Lep + Mar	63	Lep + Mar	98	Mar	36	Lep	41	Mar	44
JK6(6/1)	Lep + Mar	82	Lep + Mar	96	Lep	39	Lep	48	Lep	34
JK7(5/1)	Lep + Mar	74	Lep + Mar	97	Lep	56	Lep	89	Lep	70
JK8(4/1)	Lep	79	Lep	70	Lep	74	Lep	82	Lep	64
JK9(4/1)	Lep + Mar	92	Lep + Mar	99	Lep	52	Lep	46	Lep	44

Numbers in parentheses after the name of each jackknifed dataset indicate the number of outgroups (first number) and Leptanillinae (second number) taxa remaining after pruning heterogeneous sequences. The tree used to infer the CAT-PMSF models are indicated as: Mar–sister, Martialinae–sister; Lep–sister, Leptanillinae–sister; Lep + Mar–sister, Leptanillinae plus Martialinae–sister.

The Dayhoff-6 analyses also identified shifting support across topologies (Table 1). GTR + G analyses of eight jackknifed amino acid datasets found support for Leptanillinae plus Martialinae–sister, with the other two remaining datasets supporting Martialinae–sister. After Dayhoff-6 recoding, support for Martialinae–sister disappeared, with one dataset shifting its support to Leptanillinae–sister. In addition, two datasets that (as amino acid alignments) supported Leptanillinae plus Martialinae–sister switched support to Leptanillinae–sister upon recoding. That is, after recoding the GTR + G analyses of seven jackknifed datasets support Leptanillinae plus Martialinae–sister and three support Leptanillinae–sister. Using CAT-Poisson + G, six of the seven (recoded) jackknifed datasets supporting Leptanillinae plus Martialinae–sister under GTR + G switched support to Leptanillinae–sister. One recoded dataset continued to support Leptanillinae plus Martialinae–sister.

Posterior Predictive Analyses (PPAs), see Table S8, indicate that recoding effectively reduced compositional heterogeneity across both taxa and sites. In the PPA-MAX test, which evaluates whether alternative models can account for the across-lineage compositional heterogeneity of the data, PPA-MAX-*Z*_GTR-amino acids_ = 16.28–27.92 (Average = 20.42) is much higher than PPA-MAX-*Z*_GTR-Recoded_ = 4.11–11.53 (Average = 8.31) and PPA-MAX-*Z*_CAT-Poisson-Recoded_ = 0.03–6.27 (Average = 1.52). After recoding, CAT-Poisson + G still describes the (reduced) across-site compositional heterogeneity of the data better than GTR + G, in agreement with expectations from Giacomelli et al. (2022); PPA-DIV-*Z*_CAT-Poisson-Recoded_ = −1.38 to −0.96 (Average = −1.15), PPA-DIV-*Z*_GTR-Recoded_ = 40.06–47.88 (Average = 43.87) and PPA-DIV-*Z*_GTR-amino acids_ = 55.49–66.36 (Average = 60.42).

## Discussion

### There are three signals in ant genomes with single-gene and concatenated datasets marginally favoring different phylogenies

Our single-gene analyses point to the existence of three signals in ant genomes, corresponding to the Martialinae–sister, Leptanillinae–sister, and Leptanillinae plus Martialinae–sister hypotheses. If we use gCF or the number of genes supporting each topology as a measure of the support provided by single genes to different hypotheses, we can only conclude that the signal for Martialinae–sister is the weakest (gCF = 23.0, 331 genes). Leptanillinae plus Martialinae–sister is the most strongly supported hypothesis (gCF = 39.8, 574 genes) and Leptanillinae–sister sits between these two (gCF = 28.6, 413 genes). In addition, 8.6% of our BUSCO genes do not support any of these hypotheses. These results agree with those of Borowiec et al. (2025) which also showed that the Leptanillinae plus Martialinae–sister hypothesis is supported by the largest number of individual loci in their UCE dataset. While simple χ^2^ tests find the difference in support level provided by the gene trees to different topologies to be significant, none of the considered hypotheses is well supported. This is because if there was no phylogenetic structure in the data, we would expect each hypothesis to be supported by 33% of gene trees and sites, which is close to the best gCF we observed (gCF_Leptanillinae plus Martialinae–sister_ = 39.8). This conclusion is confirmed by results of the sCF analyses which finds an even smaller difference between the three hypotheses: sCF_Leptanillinae plus Martialinae–sister_ = 35.1, sCF_Leptanillinae–sister_ = 33.7, and sCF_Martialinae–sister_ = 33.1. These results indicate that at the single-gene level, support for alternative hypotheses is only marginally different. This is confirmed by the AU tests we performed on single-gene alignments, the results of which indicated that only 35 genes (∼2.5% of the total) have the power to discriminate between alternative hypotheses of ant relationships. Perhaps unsurprisingly, these 35 genes provide the same level of support to Leptanillinae plus Martialinae–sister and Leptanillinae–sister (16 genes out of 35), further confirming that the difference in support to these hypotheses, provided by single genes, is marginal. In these tests, Martialinae–sister continues to be the least well-supported hypothesis, being preferred only by 3 out of 35 genes.

### Across-site compositional heterogeneity is inflating support for Leptanillinae plus Martialinae–sister and Martialinae–sister

The model-test analyses that we performed on our single-gene alignments in IQTREE (using the BIC) identified an across-site compositionally heterogeneous model as best-fit for 1,388 genes, representing 96.2% of our BUSCO dataset. The relative model fit analyses performed using the BIC on the ten jackknifed datasets subsampled from the 1,442 gene concatenated alignment, agree with results from the single-gene analyses and indicate that across-site compositionally heterogeneous models fit this dataset best. Of the models implemented in IQTREE, LG + F + G + 60 invariably emerges as best-fit for our ten jackknifed datasets. Our model adequacy tests refined these results showing that CAT-PMSF + G describes the across-site compositional heterogeneity of these datasets better than LG + F + G + 60 (Average *Z*_LG + F + G + CXX_ = 46.84 and Average *Z*_CAT-PMSF_ = 2.69). Overall, these results indicate that, as suggested by Cai (2024), across-site compositional heterogeneity is an important nuisance-factor in ant phylogeny that needs to be modeled to reduce the likelihood that the recovered phylogeny might be affected by tree reconstruction artifacts. The PPAs we performed in Phylobayes using GTR + G (for the amino acid data) confirm these results (Average *Z*_GTR-amino acids_ = 20.42). Hence, we conclude that the model that best describes the across-site compositional heterogeneity of our concatenated (jackknifed) datasets is CAT-PMSF + G, and under this model support for Leptanillinae–sister is invariably found (CUBS = 87). This is different from analyses performed (for the same datasets) under LG + G and LG + F + G + C60, which support either Leptanillinae plus Martialinae–sister or Martialinae–sister, suggesting that support for these two topologies in LG + G and LG + F + G + C60 analyses is at least partially driven by poor modeling of across-site compositional heterogeneity. This conclusion is confirmed by the Bayesian CAT-Poisson + G analysis of the concatenated alignment of the 35 genes that were found to have the power to discriminate between alternative hypotheses of ant relationships in single-gene analyses, which also supports Leptanillinae–sister (PP = 1.0). The latter result is particularly interesting because the number of concatenated single-gene alignments supporting the competing Leptanillinae–sister and Leptanillinae plus Martialinae–sister in this smaller dataset was the same (16 genes in favor of both hypotheses—see above). However, support for Leptanillinae–sister under CAT-PMSF + G is not strong and none of our ten jackknifed datasets can discriminate statistically between alternative hypotheses of ant relationships when the AU test is used. We interpret the results of the AU tests performed on the concatenated datasets as confirming the conclusions derived from the results of individual gene analyses: the support for the three alternative hypotheses is only marginally different.

**Figure 3:**
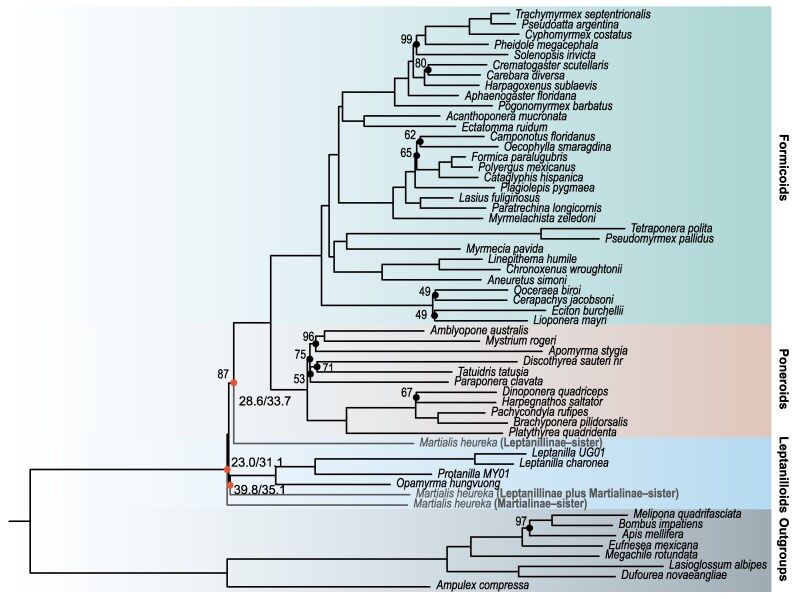
Evolutionary signals in ant genomes. This tree represents the CAT-PMSF phylogeny (Fig. 2a), with branch lengths estimated under the CAT-PMSF + G model inferred fixing the tree topology to represent the Leptanillinae–sister. The different signals for the placement of Martialinae are identified by the full red circles. The Branch lengths for Martialinae have been re-estimated under the most appropriate CAT-PMSF + G model. For example, the branch length for Martialinae when placed in the position supported by the Martialinae–sister hypothesis has been re-estimated under the CAT-PMSF + G model inferred fixing the tree topology to represent Martialinae–sister. Number at the nodes represent CUBS. For the three different hypotheses (Martialinae–sister, Leptanillinae plus Martialinae–sister, and Leptanillinae–sister), we also report gCF and sCF, indicating the relative strength of these signals. As we argue that the three signals might represent real evolutionary processes, we have represented *M. heureka* in the tree three times, with solid lines. These are not alternative possible placements for *M. heureka*, they represent what we think are real, alternative, signals in ant genomes. However, to make it clear that none of these signals might represent the phylogenetic history of this taxon, which might not be tree-like, we colored them in gray. Note that the length of the internodes identifying alternative placements of *M. heureka* have been mildly stretched to improve readibility.

All single-signal superalignments support their own tree topology and in CAT-PMSF + G analyses the Leptanillinae–sister signal (even if associated with less genes than the Leptanillinae plus Martialinae–sister signal) appears stronger. The Leptanillinae–superalignment is the only one that, in single-signal analyses, can discriminate against the other two hypotheses using the AU test and the only one providing full CUBS for its own (Leptanillinae–sister) topology. We interpret the results of the analyses of the single-signal datasets to indicate that Leptanillinae–sister is just one of three signals representing incongruent but real (ie not primarily driven by biases) evolutionary processes in ant evolution. This is because if the Leptanillinae plus Martialinae–sister and Martialinae–sister signals represented biases driven by across-site compositional attractions in the Leptanillinae plus Martialinae–superalignment and the Martialinae–superalignment, we would have expected that under CAT-PMSF + G (which models the across-site compositional heterogeneity of these datasets well) support should have been found for Leptanillinae–sister. However, this does not happen, suggesting that only a fraction of genes supporting Leptanillinae plus Martialinae–sister and Martialinae–sister might be doing so because of a tree reconstruction artifact driven by across-site compositional heterogeneity, which only magnify the support for these signals rather than creating them. We can only conclude that there seems be to three signals in the data, representing the Leptanillinae plus Martialinae–sister, Martialinae–sister, and Leptanillinae–sister hypotheses. The signals supporting Leptanillinae plus Martialinae–sister and Martialinae–sister appear exacerbated by a bias driven by across-site compositional heterogeneity. When the bias from across-site compositional heterogeneity is accounted for the signal for the Leptanillinae–sister hypothesis is the strongest, but only marginally so, and the emergence of Leptanillinae–sister in CAT-PMSF + G analyses therefore cannot be taken to imply that this topology represents must the ant phylogeny (see Fig. 3).

### Accounting for compositional heterogeneity across taxa in ant phylogeny

Borowiec et al. (2019) suggested that Leptanillinae–sister might be an artifact driven by compositional heterogeneity acting across taxa and that the correct resolution for the root of the ant phylogeny is represented by Leptanillinae plus Martialinae–sister. Leptanillinae are AT-rich in the dataset of Borowiec et al. (2019) and these authors showed that when AT-rich outgroups are included in the dataset, Leptanillinae emerges as the sister of all the other ants (Leptanillinae–sister hypothesis). Borowiec et al. (2019) showed that exclusion of AT-rich outgroups displaces Leptanillinae from the root of the ant phylogeny, leading to the emergence of trees where Leptanillinae is the sister of GC-richer Martialinae (the Leptanillinae plus Martialinae–sister hypothesis). This was an elegant experiment underpinning an interesting result.

We investigated the extent to which our individual BUSCO genes a affected by compositional heterogeneity acting across taxa. We then investigated whether there was an association between alternative hypotheses of ant relationships and the compositional heterogeneity of Leptanillinae and Martialinae. At the individual gene level, most BUSCO genes in our dataset (90.37%) do not include compositionally heterogeneous taxa. There are almost three times more genes supporting Leptanillinae–sister than there are genes including compositionally heterogeneous taxa. Accordingly, we can safely conclude that in our single-gene analyses, trees resolving Leptanillinae–sister do not represent tree reconstruction artifacts driven by compositional heterogeneity acting across taxa. This is confirmed by the observation that genes for which Leptanillinae are heterogeneous (a total of 20 out of 1,442) do not preferentially support Leptanillinae–sister. These 20 genes marginally prefer Leptanillinae plus Martialinae–sister (45%), whereby Leptanillinae–sister is recovered only by 35% of the genes where Leptanillinae are compositionally heterogeneous.

In the 10 jackknifed datasets, 9 to 13 taxa failed the χ^2^ test of compositional heterogeneity across taxa. Many of these taxa belong to either the outgroups or to Leptanillinae. This indicates that as Leptanillinae sequences are concatenated their heterogeneity adds up, and this might potentially lead to the emergence of compositional attractions in concatenated datasets, in agreement with Borowiec et al. (2019). We used taxon subsampling and data recoding to evaluate whether the signal for Leptanillinae–sister might be a compositional attraction artifact driven by compositional heterogeneity acting across lineages. After removing all the heterogeneous taxa, we found that under both LG + G and LG + F + G + C60, support for Martialinae–sister disappears. At the same time, under both models, support for Leptanillinae plus Martialinae–sister increases, with nine taxonomically reduced jackknifed datasets supporting this hypothesis instead of eight (see Tables 1 and 2). The increased support for Leptanillinae plus Martialinae–sister in these analyses is consistent with the hypothesis of Borowiec et al. (2019). However, in these analyses, support emerges also for Leptanillinae–sister, as one of the datasets that supported Leptanillinae plus Martialinae–sister changes support to Leptanillinae–sister (under both LG + G and LG + F + G + C60). This result disagrees with predictions from the hypothesis of Borowiec et al. (2019) that Leptanillinae–sister is an artifact driven by across-lineage compositional heterogeneity. The conclusion of Borowiec et al. (2019), that compositional heterogeneity acting across lineages might be a problem in ant phylogeny, is correct but from the results of the analyses presented above, it seems that the signal representing an attraction artifact might be Martialinae–sister, rather than Leptanillinae–sister. However, analyses of the same reduced datasets performed under CAT-PMSF show that support for Leptanillinae–sister decreased, despite this hypothesis remaining the most strongly supported under this model. However, in disagreement with both Borowiec et al. (2019) and with expectations based on the results we obtained in LG + G and LG + F + G + C60 analyses, it was not Leptanillinae plus Martialinae–sister that gained support as the signal for Leptanillinae–sister weakened, it was the signal for Martialinae–sister (supported in 3 out of 30 CAT-PMSF analyses after excluding heterogeneous taxa). The inconsistency in topological changes observed under different models might suggest that rather than having identified a compositional artifact, the taxon deletion experiments destabilized the phylogeny, perhaps because all but one of the Leptanillinae were removed due to their compositional heterogeneity.

PPA-MAX show, consistent with the results of Giacomelli et al. (2022), that Dayhoff-6 recoding is effective in reducing compositional heterogeneity acting across lineages. Dayhoff-6 analyses should therefore be informative of the effect of across-lineage compositional heterogeneity. Importantly, Dayhoff-6 recoding was applied to datasets where taxa were not subsampled and we cannot attribute results from these analyses to destabilizing effects potentially caused by the exclusion of all but one Leptanillinae. However, in GTR + G analyses of Dayhoff-6 recoded data, we observe the same pattern that emerged in LG + G and LG + F + G + C60 model-based analyses of taxonomically reduced datasets. That is, (i) Leptanillinae plus Martialinae–sister continues to be the most strongly supported topology, and (ii) the support for Martialinae–sister disappears, giving way to (iii) the recovery of Leptanillinae–sister (three jackknifed datasets). Under CAT-Poisson + G, six Dayhoff-6 recoded datasets that under GTR + G supported Leptanillinae plus Martialinae–sister recover Leptanillinae–sister instead, and three recoded datasets that under GTR + G already supported Leptanillinae–sister, continue to do so under CAT-Poisson + G. However, analyses of one jackknifed dataset that supported Leptanillinae plus Martialinae–sister under GTR + G continued to support this hypothesis under CAT-Poisson + G. That is similar to what we observed when analyzing the taxonomically reduced datasets under CAT-PMSF: the magnitude of support for Leptanillinae–sister is reduced in Dayhoff-6 analyses, at the least with reference to the results obtained when amino acid datasets were analyzed using CAT-PMSF. However, in contrast to the results of the CAT-PMSF analyses of taxonomically reduced datasets, we do not see the emergence of support for Martialinae–sister in the CAT-Poisson + G analyses of Dayhoff-6 recoded datasets.

We conclude that there seems to be genuine evidence to suggest that support for Martialinae–sister is at the least partially driven by across-lineage compositional heterogeneity. However, we do not find compelling evidence suggesting that Leptanillinae–sister might be an artifact driven by compositional heterogeneity acting across taxa. Finally, we agree with Borowiec et al. (2019) that there is no evidence that Leptanillinae plus Martialinae–sister might be the result of a compositional attraction driven by across-lineage compositional heterogeneity.

### Explaining incongruence in ant evolution

Many studies have attempted to reconstruct the relative relationships of Leptanillinae and Martialinae at the root at the ant phylogeny (Rabeling et al. 2008; Kück et al. 2011; Moreau and Bell 2013; Ward and Fisher 2016; Borowiec et al. 2019, 2025; Romiguier et al. 2022; Cai 2024; Boudinot and Lieberman 2025) and alternative explanations have been proposed to rationalize different trees. Borowiec et al. (2019) suggested that Leptanillinae plus Martialinae–sister might represent the true ant phylogeny and that Leptanillinae–sister represented an attraction artifact driven by compositional heterogeneity acting across lineages. Cai (2024) suggested that Leptanillinae–sister might represent the true ant phylogeny with Leptanillinae plus Martialinae–sister representing an attraction artifact driven by across-site compositional heterogeneity. Boudinot and Lieberman (2025) dismissed the conclusions of Cai (2024) suggesting that the analyses were flawed. However, their criticisms are mostly based on misunderstanding and are not supported by corroborative evidence. For example, Boudinot and Lieberman (2025) claimed that the results presented in Cai (2024) were negatively affected by the use of an alignment curation approach that was too aggressive, but did not provide any experimental evidence (eg simulations) to demonstrate this, other than indicating that the results of Cai (2024) disagree with those of Romiguier et al. (2022). The results of our study instead suggest that differences between the results of Romiguier et al. (2022), Cai (2024), and Boudinot and Lieberman (2025) more likely reflect the different ways in which these studies modeled their sequence alignments. In particular, Romiguier et al. (2022) and Boudinot and Lieberman (2025) used C20-based models and so the congruence of their results is not surprising and does not disprove the results of Cai (2024) which was based on CAT-GTR + G. Boudinot and Lieberman (2025) dismiss the CAT-GTR + G model as too complex, mirroring their criticism of Cai et al. (2022) in Boudinot et al. (2022) . Cai et al. (2024) addressed this criticism using simulations to show that the number of categories used by CAT-GTR depends on the across-site compositional heterogeneity of the data. Cai et al. (2024) showed that CAT-GTR does not even overparameterize when used to analyze data that exhibits no across-site compositional heterogeneity. This is because, under such conditions, CAT-GTR instantiates a model where the median number of site-frequency categories (and hence compositional vectors) across generations (after convergence) is equal to one. Under such a model, the sites in the alignment can only be assigned to one category, and a CAT-GTR model with one category equates to a standard (across-site compositionally homogeneous) GTR model. Hence, CAT-GTR models are not necessarily more complex than standard across-site compositional homogeneous models; to say as much without providing evidence of how their presumedly excessive complexity negatively affected Cai (2024) study is not a valid criticism. Indeed, our parametric bootstrap analyses indicate that, to the contrary, C20-based models such as those of used in Romiguier et al. (2022) and Boudinot and Lieberman (2025) fail to adequately account for the across-site compositional heterogeneity of ant phylogenomic datasets. As we have discussed, Boudinot and Lieberman (2025) go further in their criticism of Cai (2024), arguing that CAT-based models do not account for compositional heterogeneity, but this was achieved by redefining compositional heterogeneity in an incomplete way so that it reflected only across-lineage compositional heterogeneity. Mechanistically, CAT-based models are rate models. However, they assume sites to have different amino acid equilibrium frequencies, which they account for using a variable number of site-frequency profiles that are characterized by different amino acid compositional vectors. Accordingly, phenomenologically, they account for site-specific amino acid preferences, ie across-site compositional heterogeneity. Hence, contrary to what stated by Boudinot and Lieberman (2025), Cai (2024) analyses accounted for compositional heterogeneity. However, Boudinot and Lieberman (2025) are correct when stating that Cai (2024) did not address across-lineage compositional heterogeneity and this may be a shortcoming given the results of Borowiec et al. (2019).

Our study addressed both across-site and across-lineage compositional heterogeneity in attempting to resolve ant phylogeny. We investigated whether population-level phenomena such as introgression and incomplete lineage sorting might have resulted in the presence of multiple evolutionary signals in the data, which might also drive phylogenetic incongruence. Our results are in better agreement with those of Cai (2024) than with those of Borowiec et al. (2019), Romiguier et al. (2022), and Boudinot and Lieberman (2025). This is because we found evidence indicating that support for Leptanillinae plus Martialinae–sister and Martialinae–sister in our ten jackknifed amino acid datasets is partially driven by across-site compositional heterogeneity, with support for Martialinae–sister also being partially driven by compositional heterogeneity acting across lineages. At the same time, contrary to Borowiec et al. (2019), we did not find compelling evidence that across-lineage compositional heterogeneity might be magnifying the support for Leptanillinae–sister, despite confirming that Leptanillinae are mostly compositionally heterogeneous in concatenated datasets (Borowiec et al. 2019).

However, we suggest that our results should not be interpreted to indicate that Leptanillinae are the sister of all the other ants. This is because our single-signal datasets suggest that there seem to be three genuine evolutionary signals in ant genomes, corresponding to Leptanillinae–sister, Leptanillinae plus Martialinae–sister, and Martialinae–sister, even if the last two seem to be exacerbated by the presence of compositional biases. ASTRAL has been proved to be consistent under the multispecies coalescent. Accordingly, it could be argued that in the presence of multiple signals in the data, our ASTRAL topology might be a better estimate of the true ant phylogeny. However, ASTRAL can only be expected to be consistent under the assumption that the gene trees it combines are an accurate representation of the frequency with which different gene trees support different ant phylogenies (Xi et al. 2015), which is unlikely to be the case in our dataset, where the support for Leptanillinae plus Martialinae–sister seems to be exacerbated by across-site compositional heterogeneity.

We therefore conclude that three phylogenetic signals exist within ant genomes. Among them, the Leptanillinae plus Martialinae–sister signal seems to be partially driven by compositional heterogeneity acting across sites. The Martialinae–sister signal is the weakest and it seems partially driven by compositional heterogeneity acting across both sites and taxa. The signal for Leptanillinae–sister seems less affected by compositional biases than the other two. However, all three signals seem to represent the signature of genuine evolutionary processes (eg phylogeny, introgression, and incomplete lineage sorting), with the compositional biases magnifying, rather than generating, the Leptanillinae plus Martialinae–sister and the Martialinae–sister signals. Building phylogenies is not sufficient to identify which one of the three signal (if any) is representative of the ant phylogeny. Accordingly, no published study, including our own, can be said to have provided the evidence needed to solve this problem (irrespective of their relative strength) of these signals in their respective analyses. To understand which one of the three tree topologies represents phylogenetic signal and what the other signals represent (introgression or incomplete lineage sorting), other types of analyses are necessary (eg Feng et al. 2022) and it is well possible that the fundamental divergence in ant phylogeny might be unresolvable and unknowable (Fig. 3).

Nevertheless, we hope that our study might represent a useful starting point for authors interested in understanding the incongruent phylogenetic signals harbored by ant (and other) genomes, rather than simply trying to infer a tree from data that might not necessarily have evolved from a strictly bifurcating evolutionary history.

## Materials and methods

### Data collection, genomes assembly, and single-copy ortholog identification

We started with a dataset of 101 species (91 ants and 10 outgroups—see Table S9 for a list of taxa and accession numbers) which includes the 82 taxa in Romiguier et al. (2022), to which we added the genomes and transcriptomes for 19 taxa that were available when we started our study (2023 May). The new genomes and transcriptomes were trimmed and assembled using BBNorm and SPAdes v3.15.0 (Prjibelski et al. 2020). We used the 5,991-gene Hymenoptera dataset in BUSCO v5.4.7 (Manni et al. 2021) to identify single-copy orthologs from our 101-taxon dataset (95 genomes and 6 transcriptomes) (see Table S10 for BUSCO scores).

### Alignment curation and gene selection

All 5,991 BUSCO genes were trimmed by Prequal v1.02 (Whelan et al. 2018). They were then aligned in Mafft v7.429 (Katoh et al. 2005) with the L-INS-i algorithm. The alignments were further trimmed using BMGE v1.12 (Criscuolo and Gribaldo 2010), default options.

An initial set of 5,991 gene trees were generated using the LG + G model in IQTREE v2.2.0.3 (Le and Gascuel 2008; Minh et al. 2020). These gene trees were used to identify and remove long branched taxa using TreeShrink v1.3.9 (Mai and Mirarab 2018). At the end of this process, six gene families included less than four taxa and were excluded from further analyses. The remaining 5,985 BUSCO genes were realigned and used to generate a new set of gene trees (using LG + G) in IQTREE v2.2.0.3. Clan-Check (Siu-Ting et al. 2019) was used on these trees to identify genes that failed to correctly split (i) the ingroups from the outgroups and (ii) the 20 poneroid species from the other taxa in our dataset. These filters are effective since the monophyly of these groups (ants and poneroids) is well supported and so genes that cannot resolve these groups are likely affected by hidden paralogy or other sources of phylogenetic error (eg Siu-Ting et al. 2019; Mulhair et al. 2022; Pisani et al. 2022; McCarthy et al. 2023). Clan-Check removed 3,736 gene families, leaving us with a dataset of 2,249 BUSCO genes. Since our goal is to understand the instability of *M. heureka* with reference to previous analyses (Romiguier et al. 2022; Cai 2024; Borowiec et al. 2025; Boudinot and Lieberman 2025), we further subsampled the BUSCO genes to make sure that all retained gene families included (i) *M. heureka*, (ii) at least one species from Leptanillinae, (iii) at least one outgroup species, and (iv) at least one species from the poneroid and formicoid clades. After this final subsampling step, our dataset encompassed 1,442 BUSCO genes (see Table S2 for a list of genes).

### Gene tree inference, taxon subsampling, and ASTRAL analyses

Our 101-taxon, 1,442 gene dataset (1,105,819 amino acid positions) can be easily analyzed using fast heuristic approaches such as ASTRAL. However, it is not possible to analyze such a large dataset as a concatenated superalignment using the infinite mixture models accounting for across-site compositional heterogeneity that have featured prominently in recent debate over ant phylogeny (Cai 2024; Boudinot and Lieberman 2025). Accordingly, we performed an analysis of our full dataset with ASTRAL v5.7.8 and then subsampled the taxa to generate a more manageable 55-taxon, 1,442-gene dataset. To subsample the dataset taxonomically, we removed taxa nested highly within the poneroid and formicoid clades that can be expected, a priori, to have little influence on the relationships at the root of the ant phylogeny. To ensure that taxon subsampling did not negatively affect the signal in our dataset, we performed a second ASTRAL analysis using the 55-taxon dataset. For both analyses, we used gene trees generated under the best-fitting model identified in IQTREE v2.0.6 using the BIC (Kalyaanamoorthy et al. 2017). Model selection tested both across-site compositionally homogeneous models (eg LG + G) and across-site compositionally heterogeneous models available in IQTREE, ie the empirical mixture models with fixed numbers of site-frequency categories of Le et al. (2008). For the latter, we specified and tested models with the form: LG + G + F + CXX—where XX is either 10, 20, 30, 40, 50, or 60. Once the two ASTRAL trees were generated, we compared them to make sure that they did not differ from each other in any significant way (Fig. S1). The congruence of the 101- and the 55-taxon trees was considered evidence that subsampling taxa did not distort the phylogenetic signal in the data.

### Gene- and site-discordance analyses and the support of gene trees for alternative hypotheses of ant phylogeny

We first concatenated our 1,442 genes to generate a superalignment that we analyzed using LG + G in IQTREE. The LG + G tree recovered Leptanillinae plus Martialinae–sister (Figure S3a) and we used Mesquite v3.81 (Maddison and Maddison 2023) to modify the Leptanillinae plus Martialinae–sister tree in Fig. S3a and generate two more tree topologies, one representing the Leptanillinae–sister hypothesis and one representing the Martialinae–sister hypothesis (Fig. S3b and c). The only difference between the three tree topologies is represented by the placement of *M. heureka.* For the three hypotheses of ant relationships and our 1,442 gene trees, we estimated gCF and sCF in IQTREE v2.4.0.

Single-gene alignments are short and individual gene trees can be affected by errors (Xi et al. 2015). Accordingly, they might agree with one of the three competing hypotheses of ant relationships by chance. To address this problem, we investigated how many of the 1,442 genes in our dataset could discriminate statistically between Leptanillinae plus Martialinae–sister, Leptanillinae–sister, and Martialinae–sister, using the AU test (Shimodaira 2002).

### Gene concatenation analyses: comparing across-site compositionally homogeneous and heterogeneous models

We inferred species trees from the concatenation of 1,442 genes under LG + G and using the best-fitting empirical mixture model with fixed number of site-frequency categories (ie a model with the form: LG + F + G + CXX), as identified (using the BIC) by Modelfinder in IQTREE v2.4.0, and with the recently developed CAT-PMSF (Szánthó et al. 2023) method, which facilitates porting good approximations of dataset-specific, CAT-based models inferred in Phylobayes (Lartillot et al. 2013) to ML. Finally, we generated a concatenation from the subset of genes which could discriminate between alternative hypotheses of ant relationships based on the AU test (irrespective of what trees they preferred) and analyzed this much shorter (35 genes and 40,647 amino acid sites; see results) dataset using the CAT-Poisson + G model in Phylobayes MPI v1.9 (Lartillot et al. 2013).

CAT-PMSF (Szánthó et al. 2023) uses Bayesian analysis performed under a fixed tree topology in Phylobayes (Lartillot et al. 2013) to infer a site-frequency profile under an infinite mixture model, in our case CAT-Poisson + G. The site-frequency profile is then exported to IQTREE where an unconstrained ML tree search is performed (eg Szánthó et al. 2023; Giacomelli et al. 2024; Redmond 2024). In this way, CAT-PMSF ports approximations of CAT-based models that have the potential to better describe the across-site compositional heterogeneity of concatenated alignments than empirical mixture models with fixed numbers of categories to ML, avoiding convergence problems that frequently arise when model optimization and tree-search are concomitantly performed in a Bayesian framework (Giacomelli et al. 2024). However, inferring a dataset-specific site-frequency profile in Phylobayes, even under a fixed topology, proved impossible for our full 1,442-gene concatenated dataset (1,105,819 amino acid positions). Accordingly, we used the jackknife to generate ten smaller datasets of 50,000 sites each. For each jackknifed dataset, we used the PMSF procedure to infer a dataset-specific site-frequency profile (under CAT-Poisson + G) in Phylobayes MPI v1.9 (Lartillot et al. 2013). Giacomelli et al. (2024) showed that the fixed topology used to infer a CAT-PMSF profile might bias the result of the subsequent ML analysis mildly, and so for each jackknifed dataset we inferred three site-frequency profiles, fixing the tree topology to represent each of the three competing hypotheses of ant relationships. For each jackknifed dataset and tree topology, two independent chains were run and convergence was assessed using “tracecomp.” We used a burnin of 2,500 generations and made sure that ESS > 100 and reldiff < 0.3 for all parameters (see Phylobayes manual). As the tree topologies were fixed, there was no need to test whether topological convergence was reached using “bpcomp.” The site profiles were extracted using the “readpb –ss” command, sampling 100 points of the posterior parameter space; these were converted into a format that can be read by IQTREE using “convert-site-dists.py” (Szánthó et al. 2023). ML CAT-PMSF analyses were then performed in IQTREE, combining our dataset-specific site-frequency profiles, a Poisson process of amino acid substitution, and using a Gamma distribution to model across-site rate variation (CAT-PMSF + G). As we analyzed 10 jackknifed datasets using three different CAT-PMSF + G models, we performed a total of 30 ML CAT-PMSF analyses. Following Giacomelli et al. (2024), the final CAT-PMSF + G tree was reconstructed summarizing the ultrafast bootstrap trees from each of the 30 CAT-PMSF + G analyses into a single consensus tree. Support values for trees inferred pooling results from multiple jackknifed datasets are here referred to as CUBS (Cumulative Ultrafast Bootstrap Support). To allow for a fair comparison of CAT-PMSF + G results against results inferred using other models, the same ten jackknifed datasets were also analyzed using LG + G, and under their best-fitting empirical mixture model with fixed number of site-frequency categories (LG + F + G + CXX—selected for each jackknifed dataset using the BIC in IQTREE). For the LG + G and LG + F + G + CXX models, CUBS values were calculated following the procedure described above for CAT-PMSF + G.

In analyses of sequence data, it is customary to prefer the tree inferred under the best-fitting model (eg Tihelka et al. 2021), estimated using a relative fit test such as the BIC (see also above). Testing the relative fit of CAT-PMSF + G against LG + G and LG + F + G + CXX using BIC is not possible because the number of parameters of the CAT model in Phylobayes is not carried over to the ML analyses (Giacomelli et al. 2024). However, absolute fit tests (model adequacy tests) can still be used to evaluate how CAT-PMSF + G, LG + G, and LG + F + G + CXX describe key properties of the data (Bollback 2002; Lartillot et al. 2007; Shepherd and Klaere 2018). The core area of disagreement between Cai (2024) and Boudinot and Lieberman (2025) relates to the relative ability of infinite mixture models and empirical mixture models with fixed numbers of categories to model across-site compositional heterogeneity. Accordingly, we used model adequacy tests of amino acid diversity (measuring the mean amino acid diversity per site of our datasets; see Phylobayes manual), as implemented in the ML framework using Parametric Bootstrap (Giacomelli et al. 2024) to compare the fit of LG + G, CAT-PMSF + G, and the best-fitting LG + F + G + CXX model (which for all our jackknifed datasets was LG + F + G + C60).

In model adequacy tests, the average amino acid (site) diversity of the empirical data is compared with that of datasets simulated under the tested model. If a model describes the data adequately, the value measured for the real dataset should fall within the distribution of the values from the simulated data. We performed model adequacy tests for all three CAT-PMSF models (for each of the ten jackknifed datasets) and compared the results against those obtained using the LG + G and LG + F + G + C60. We consider a model as adequately fitting the data when −2 < *Z*-score < 2 (Giacomelli et al. 2022, 2024).

We tested whether the jackknifed datasets statistically discriminate between alternative hypotheses of ant relationships using the AU test, performed under the model with the best absolute fit, as estimated from the parametric bootstrap analyses (ie CAT-PMSF + G). For each jackknifed dataset, we performed a test under each of the three CAT-PMSF + G models. Accordingly, the three hypotheses of ant relationships were tested three times on each one of the 10 jackknifed datasets, resulting in a total of 30 AU tests. We used the Bonferroni correction to adjust the significance level, Bonferroni corrected *P*-value (α-corrected) = 0.05/30 = 0.0016.

### Testing whether the three hypotheses of ant relationships represent evolutionary processes or biases driven by across-site compositional heterogeneity

To evaluate whether the three alternative hypotheses of ant relationships are underpinned by real evolutionary processes or biases (driven by across-site compositional heterogeneity), we tested the strength of the signals in our dataset. We partitioned the 1,442 alignments into three groups depending on which one of the three hypotheses of ant relationships they supported in single-gene analyses performed under their best-fitting model. From each of these three groups of alignments, we generated a concatenated dataset (the Leptanillinae–superalignment: 413 genes—see results; the Leptanillinae plus Martialinae–superalignment: 574 genes—see results; and the Martialinae–superalignment: 331 genes—see results), which we refer to as the “single-signal” datasets because they merge gene trees supporting the same phylogeny (ie they are homogeneous with reference to the phylogenetic tree they support). Each single-signal dataset was analyzed using CAT-PMSF + G, LG + G, and LG + F + G + C60. For these analyses, we used the same protocol described in the previous section but generated only three jackknifed datasets as these superalignments are shorter than the 1,442-gene superalignment. We investigated the strength of the signals in the jackknifed datasets testing what tree they support and whether they could discriminate between alternative hypotheses of ant relationships using the AU test (under each one of the three CAT-PMSF models). Because the AU tests were used as a means of investigating the strength of the signal in the data, rather than to explicitly reject hypotheses, we did not use Bonferroni corrections for multiple testing.

We used parametric bootstrap (see above) to test the model adequacy of LG + G, LG + F + G + C60, and CAT-PMSF for these superalignments. We only tested one randomly selected jackknifed dataset. Since Modelfinder always prefers LG + F + G + C60 in the 1,442-gene jackknife analysis, for analyses to be performed under LG + F + G + CXX we used LG + F + G + C60 without completing further analyses of model-fit.

We reasoned that if the signal in a single-signal dataset (eg the Leptanillinae plus Martialinae–superalignment) represents a bias driven by across-site compositional heterogeneity, we should observe a switch in support to a different hypothesis (eg Leptanillinae–sister) in CAT-PMSF + G analyses. This is because CAT-PMSF + G fits across-site compositionally heterogeneous models better than LG + G and LG + F + G + C60 (Giacomelli et al. 2024). On the other hand, if Leptanillinae plus Martialinae–sister represents a real evolutionary process (ie phylogeny, introgression, or incomplete lineage sorting) in the Leptanillinae plus Martialinae–superalignment, we should expect the Leptanillinae plus Martialinae–superalignment to continue to support Leptanillinae plus Martialinae–sister also under CAT-PMSF + G, as in this case there should be no compositional attraction to correct.

### Testing the influence of across-lineage compositional heterogeneity on ant phylogeny

It has been suggested that ant phylogeny might be negatively affected by compositional heterogeneity acting across lineages (Borowiec et al. 2019; Boudinot and Lieberman 2025). Accordingly, we investigated the effect of this form of heterogeneity on our results. In the individual gene analyses, we extracted a list of all taxa that did not pass the χ^2^ test of compositional homogeneity (*P*-value < 5%) from the 1,442 IQTREE output log files and investigated whether alignments with a greater number of taxa not passing the χ^2^ test of compositional heterogeneity preferentially supported Leptanillinae–sister. With our concatenated datasets, we would have ideally used models that account for across-lineage compositional heterogeneity (eg the Node Discrete Compositionally Heterogeneous [NDCH] model; Foster 2004). However, these models are computationally very expensive and its application is likely intractable given our datasets. Instead, we used alternative approaches that have been designed to reduce the effect of across-lineage compositional heterogeneity or to test its effects. First, from our ten jackknifed datasets, we identified and removed all taxa whose *P*-value < 10% in χ^2^ tests of compositional heterogeneity (performed in IQTREE v2.4.0). We then analyzed these taxonomically reduced datasets using LG + G, LG + F + G + C60, and CAT-PMSF + G. This approach followed by Borowiec et al. (2019). As above, each CAT-PMSF + G analysis used three models inferred using the three competing hypotheses of ant phylogeny. We then compared the trees inferred from the taxonomically reduced datasets against those inferred using the full datasets. Second, we recoded the ten jackknifed datasets (full taxon sampling) using Dayhoff-6 (eg Feuda et al. 2017). As clearly illustrated in Giacomelli et al. (2022), and contrary to Hernandez and Ryan (2021) who achieved different conclusions because their simulated datasets were too short and hence uninformative upon recoding, Dayhoff-6 recoding is effective at reducing compositional heterogeneity across both lineages and sites. Furthermore, because recoded datasets have a reduced state-set, they achieve better levels of convergence in Bayesian analyses faster (Giacomelli et al. 2022). Accordingly, for the ten recoded datasets, we performed Bayesian phylogenetic analyses under GTR + G and CAT-Poisson + G in Phylobayes MPI v1.9, rather than using the CAT-PMSF + G approximations of CAT-Poisson + G. For comparison, we also used Phylobayes MPI v1.9 to infer trees from the original amino acid datasets under GTR + G, as with this model good levels of convergence are often achieved also with amino acid datasets. Good levels of convergence were achieved for all analyses (ESS > 100, reldiff < 0.3, and maxdiff < 0.3).

For all the datasets and models for which we performed Bayesian analyses (ten Dayhoff-6 datasets analyzed under CAT-Poisson + G and GTR + G, and ten amino acid datasets analyzed under GTR + G), we used Posterior Predictive Analysis (PPA; as implemented in Phylobayes MPI v1.9) to test whether recoding the data reduced compositional heterogeneity. To do this, we compared PPA results obtained for the Dayhoff-6 and amino acid datasets under GTR + G. With the recoded data, we also compared PPA results for the two models (GTR + G and CAT-Poisson + G), to evaluate which one better described the across-site and across-lineage compositional heterogeneity remaining in the data after recoding. In this context, it is important to note that Dayhoff-6 reduces, but does not eliminate, the heterogeneity in the data (Giacomelli et al. 2022) and the residual heterogeneity still needs to be adequately modeled. Nevertheless, by reducing heterogeneity, Dayhoff-6 recoding allows available models to more adequately describe the data (Giacomelli et al. 2022). The statistics used to test for across-site compositional heterogeneity was the mean amino acid diversity per site (the same statistics we used in our parametric bootstrap analyses)—PPA-DIV, while the statistic used to test across-lineage compositional heterogeneity was the maximum square deviation between global and taxon-specific empirical frequencies—PPA-MAX (see Phylobayes manual).

## Supplementary Material

msag058_Supplementary_Data

## Data Availability

All infill and output files are available at 10.6084/m9.figshare.30920237.
