# Supplementary figures and images for: Conflict in ant phylogeny results from complex interaction between multiple evolutionary signals and tree reconstruction artifacts
Source: Mol Biol Evol. 2026 Mar 7;43(4):msag058. doi: 10.1093/molbev/msag058 (PMC13048905; doi:10.1093/molbev/msag058)

A

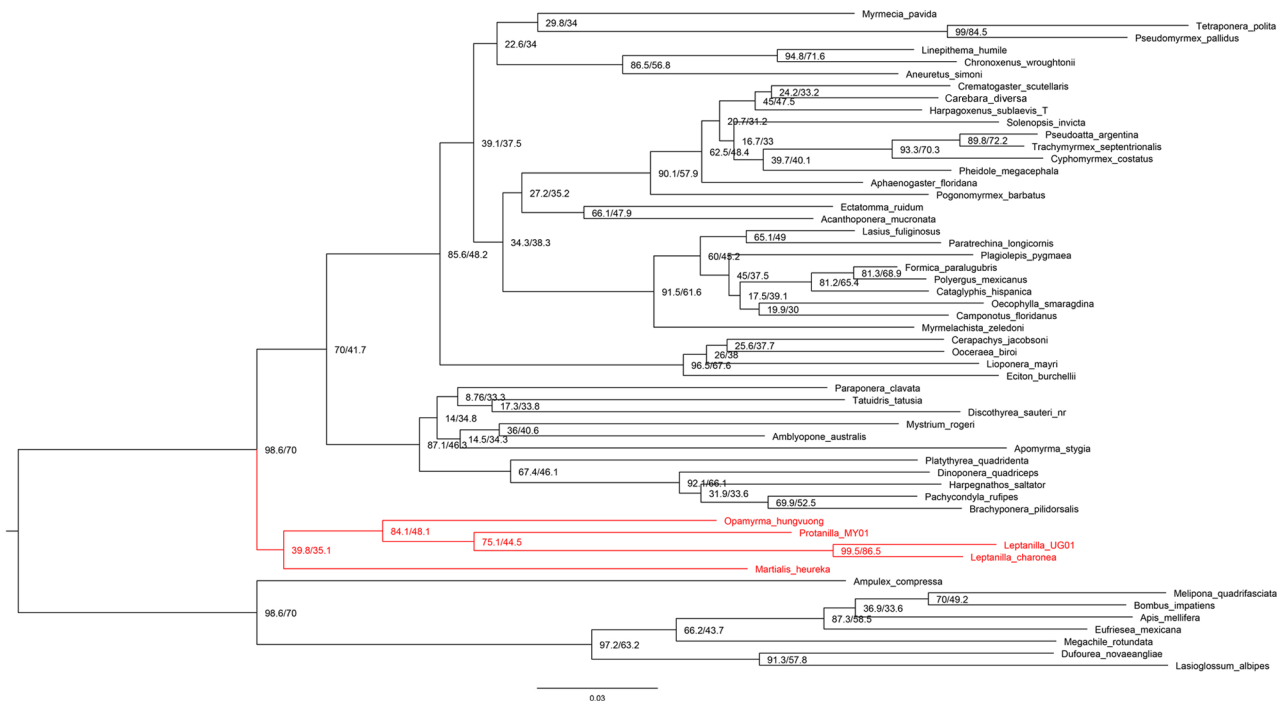

B

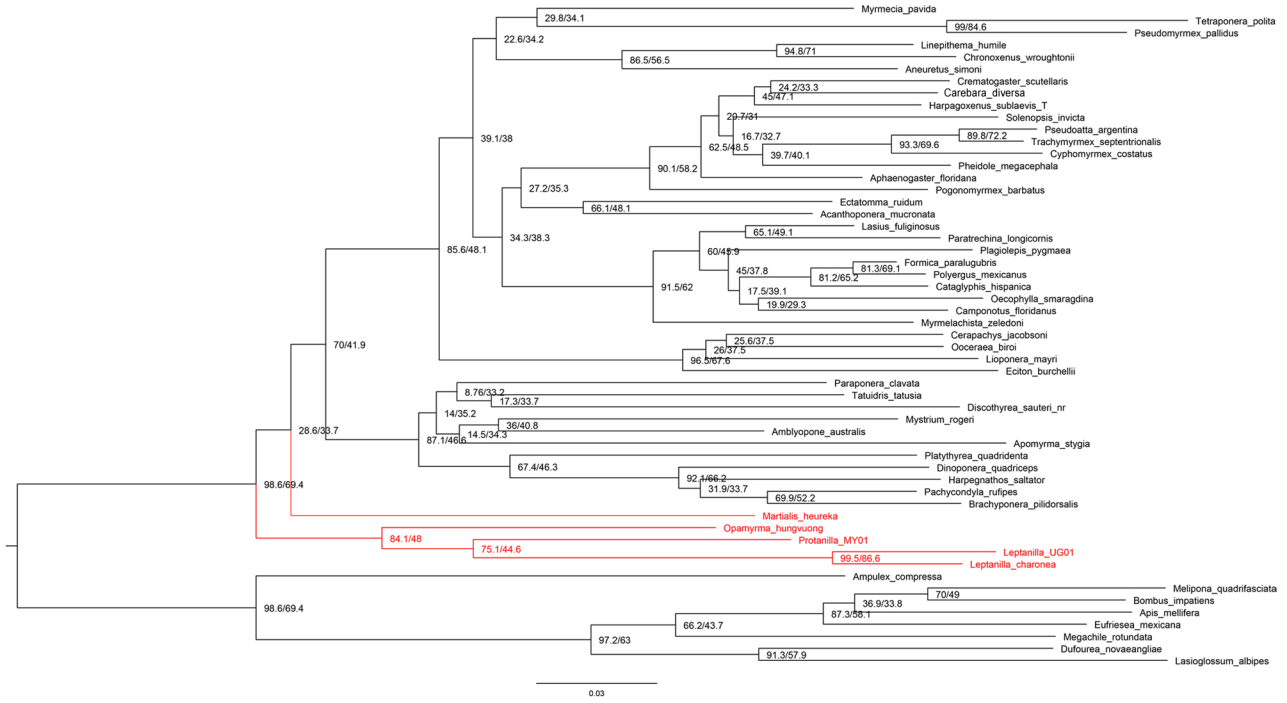

C

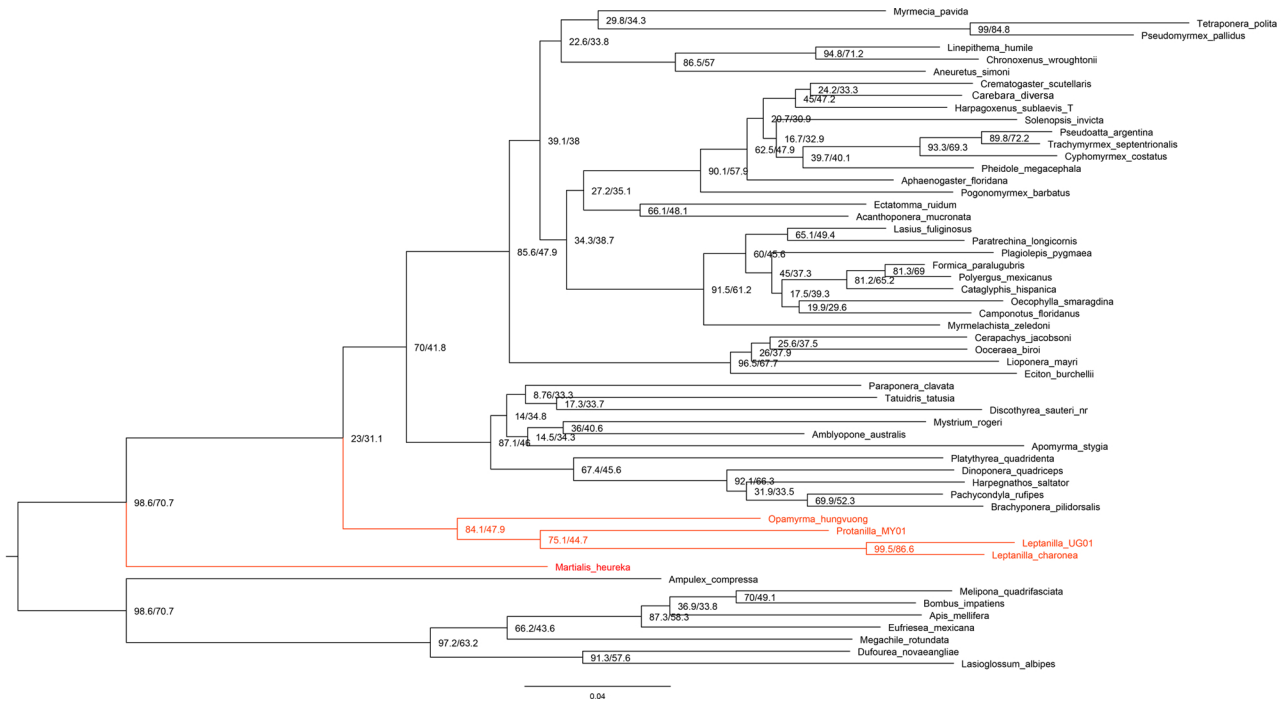

Supplement: msag058_Supplementary_Data [file msag058_supplementary_data.zip › FigS2_synonym_revised.pdf]

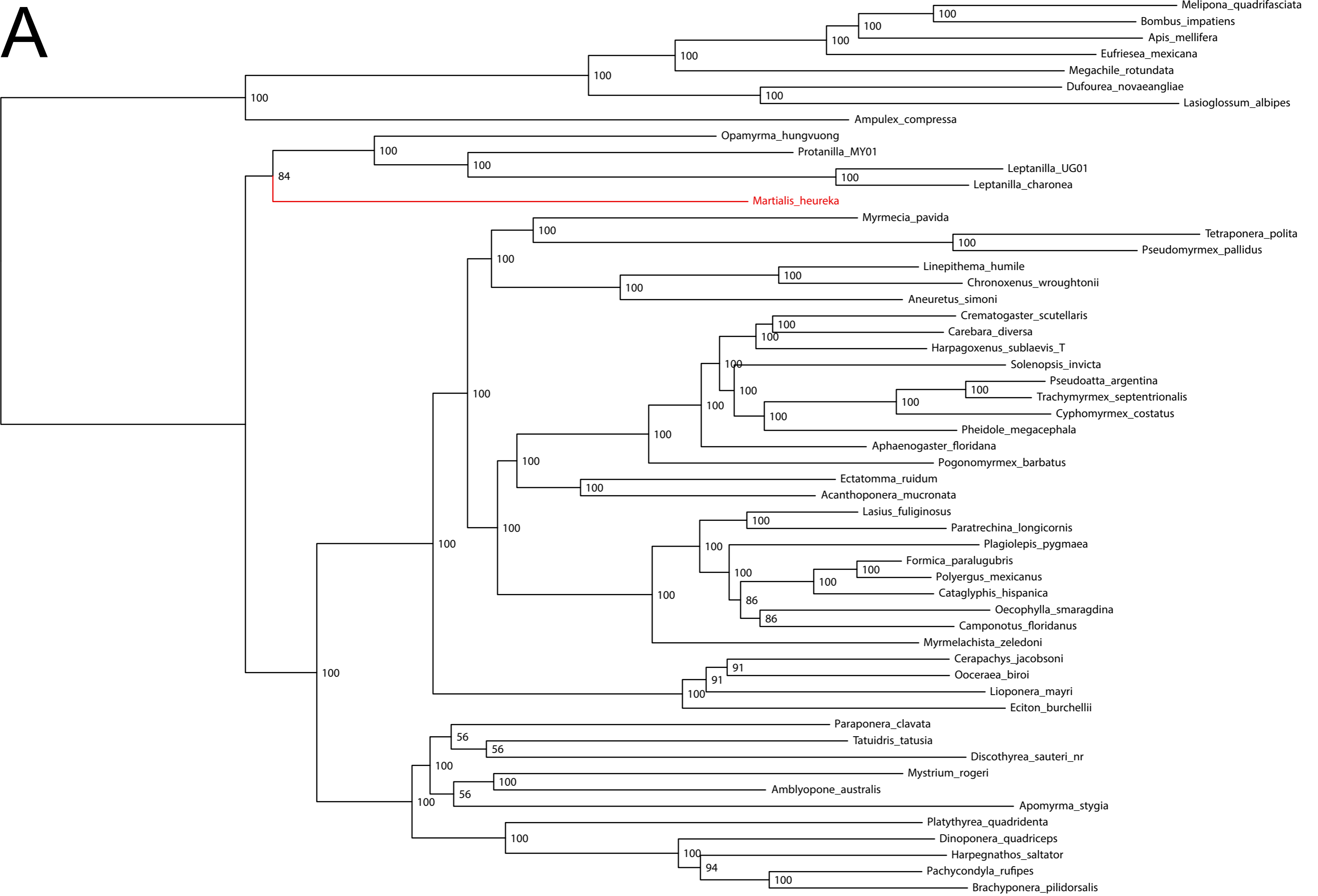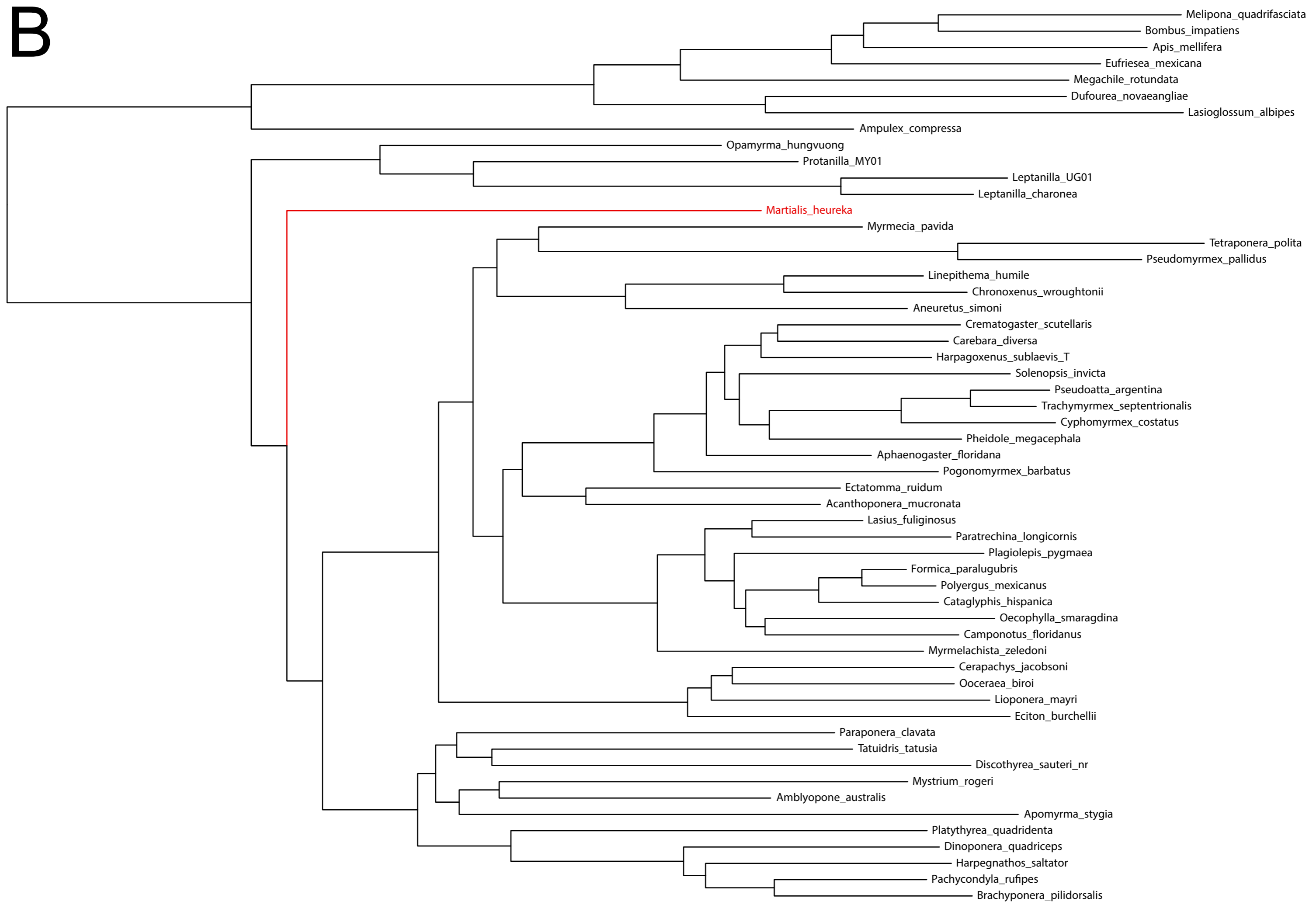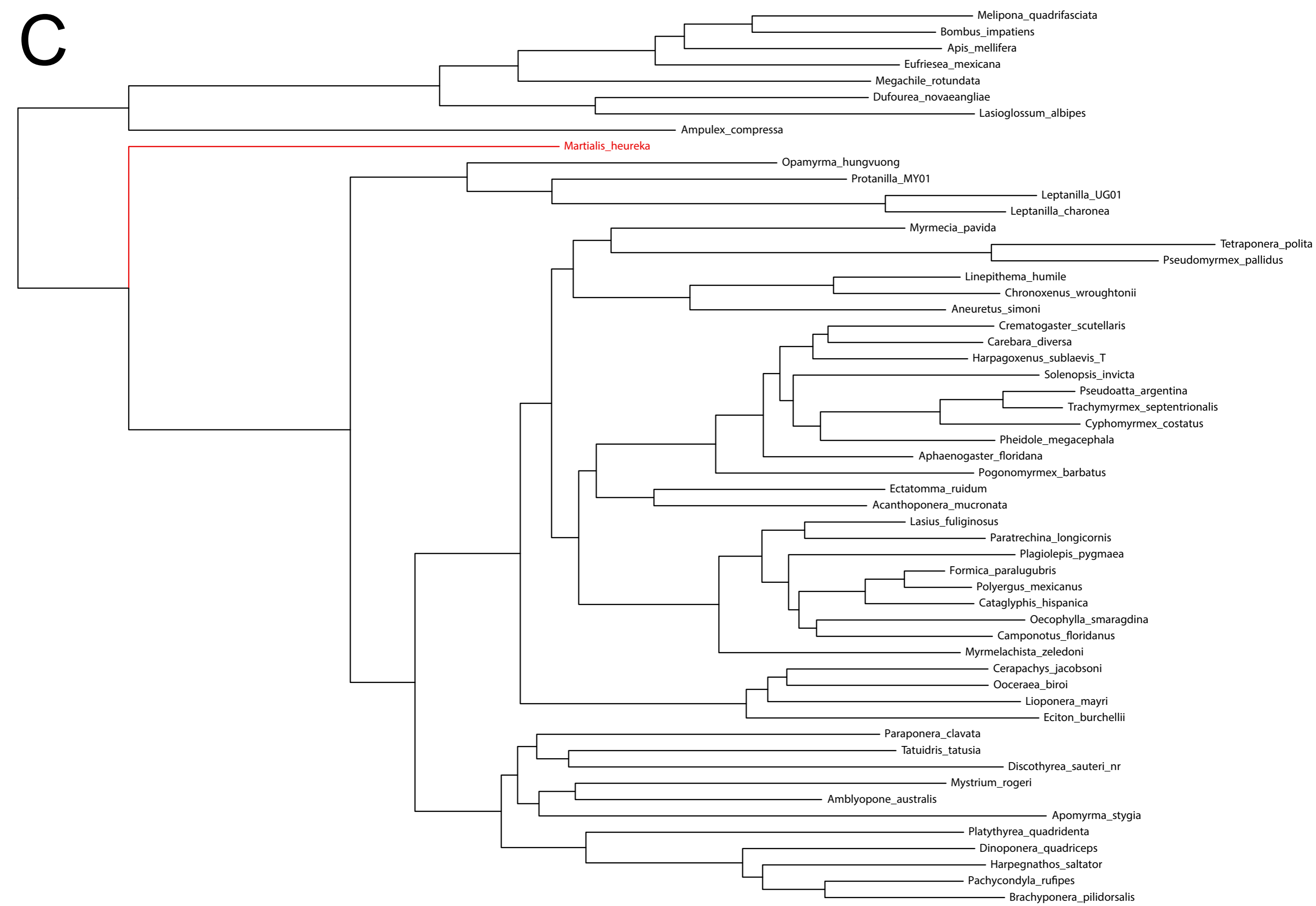

Supplement: msag058_Supplementary_Data [file msag058_supplementary_data.zip › FigS3_synonym_revised.pdf]

A

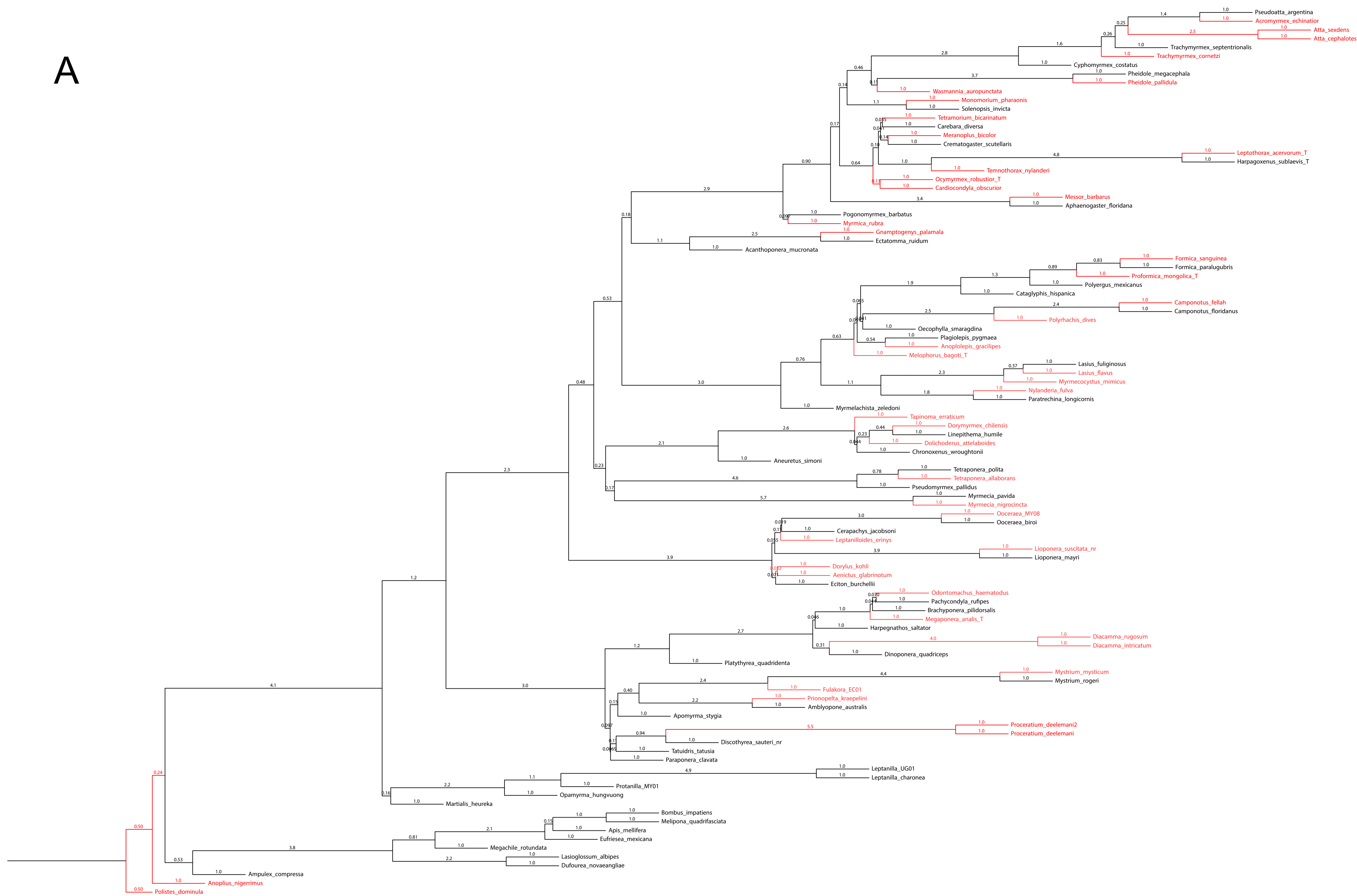

B

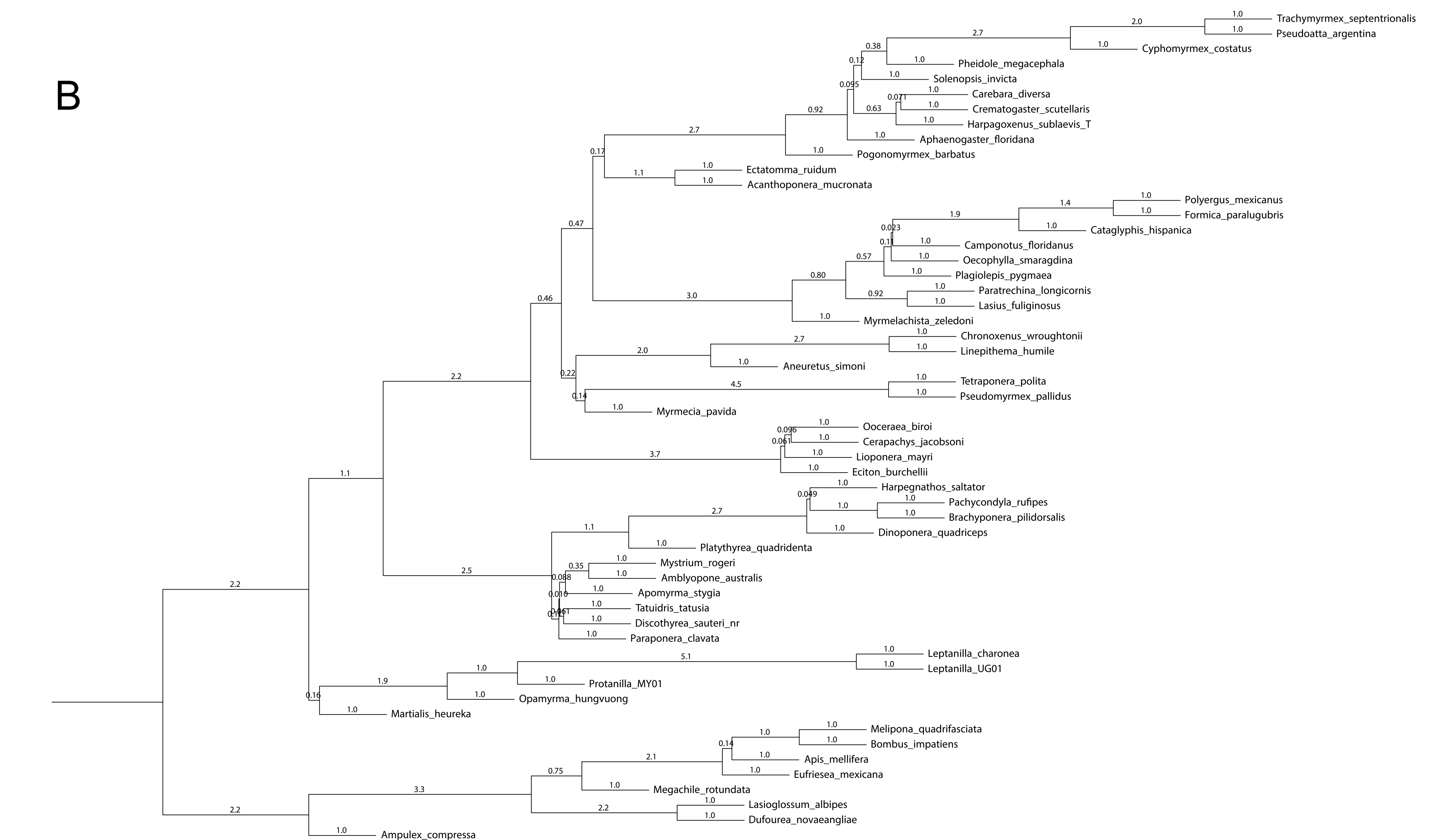

Supplement: msag058_Supplementary_Data [file msag058_supplementary_data.zip › FigS1_synonym_revised.pdf]
